# Multi-step ahead forecasting of electrical conductivity in rivers by using a hybrid Convolutional Neural Network-Long Short-Term Memory (CNN-LSTM) model enhanced by Boruta-XGBoost feature selection algorithm

**DOI:** 10.1038/s41598-024-65837-0

**Published:** 2024-07-01

**Authors:** Masoud Karbasi, Mumtaz Ali, Sayed M. Bateni, Changhyun Jun, Mehdi Jamei, Aitazaz Ahsan Farooque, Zaher Mundher Yaseen

**Affiliations:** 1 Canadian Centre for Climate Change and Adaptation, University of Prince Edward Island, St Peters Bay, PE Canada; 2 Water Engineering Department, Faculty of Agriculture, University of Zanjan, Zanjan, Iran; 3 UniSQ College, University of Southern Queensland, Springfield Campus, QLD 4301 Australia; 4 Department of Civil, Environmental and Construction Engineering and Water Resources Research Center, University of Hawaii at Manoa, Honolulu, HI 96822 USA; 5 Department of Civil and Environmental Engineering, College of Engineering, Chung-Ang University, Seoul, Republic of Korea; 6 Faculty of Civil Engineering and Architecture, Shahid Chamran University of Ahvaz, Ahvaz, Iran; 7 Faculty of Sustainable Design Engineering, University of Prince Edward Island, Charlottetown, PE C1A4P3 Canada; 8 Civil and Environmental Engineering Department, King Fahd University of Petroleum & Minerals, 31261 Dhahran, Saudi Arabia; 9 New Era and Development in Civil Engineering Research Group, Scientific Research Center, Al-Ayen University, Thi-Qar, Nasiriyah, 64001 Iraq

**Keywords:** Electrical conductivity, Time series forecasting, Boruta feature selection, Convolutional neural network, Long short-term memory, Environmental sciences, Hydrology, Engineering

## Abstract

Electrical conductivity (EC) is widely recognized as one of the most essential water quality metrics for predicting salinity and mineralization. In the current research, the EC of two Australian rivers (Albert River and Barratta Creek) was forecasted for up to 10 days using a novel deep learning algorithm (Convolutional Neural Network combined with Long Short-Term Memory Model, CNN-LSTM). The Boruta-XGBoost feature selection method was used to determine the significant inputs (time series lagged data) to the model. To compare the performance of Boruta-XGB-CNN-LSTM models, three machine learning approaches—multi-layer perceptron neural network (MLP), K-nearest neighbour (KNN), and extreme gradient boosting (XGBoost) were used. Different statistical metrics, such as correlation coefficient (R), root mean square error (RMSE), and mean absolute percentage error, were used to assess the models' performance. From 10 years of data in both rivers, 7 years (2012–2018) were used as a training set, and 3 years (2019–2021) were used for testing the models. Application of the Boruta-XGB-CNN-LSTM model in forecasting one day ahead of EC showed that in both stations, Boruta-XGB-CNN-LSTM can forecast the EC parameter better than other machine learning models for the test dataset (R = 0.9429, RMSE = 45.6896, MAPE = 5.9749 for Albert River, and R = 0.9215, RMSE = 43.8315, MAPE = 7.6029 for Barratta Creek). Considering the better performance of the Boruta-XGB-CNN-LSTM model in both rivers, this model was used to forecast 3–10 days ahead of EC. The results showed that the Boruta-XGB-CNN-LSTM model is very capable of forecasting the EC for the next 10 days. The results showed that by increasing the forecasting horizon from 3 to 10 days, the performance of the Boruta-XGB-CNN-LSTM model slightly decreased. The results of this study show that the Boruta-XGB-CNN-LSTM model can be used as a good soft computing method for accurately predicting how the EC will change in rivers.

## Introduction

**Figure 1:**
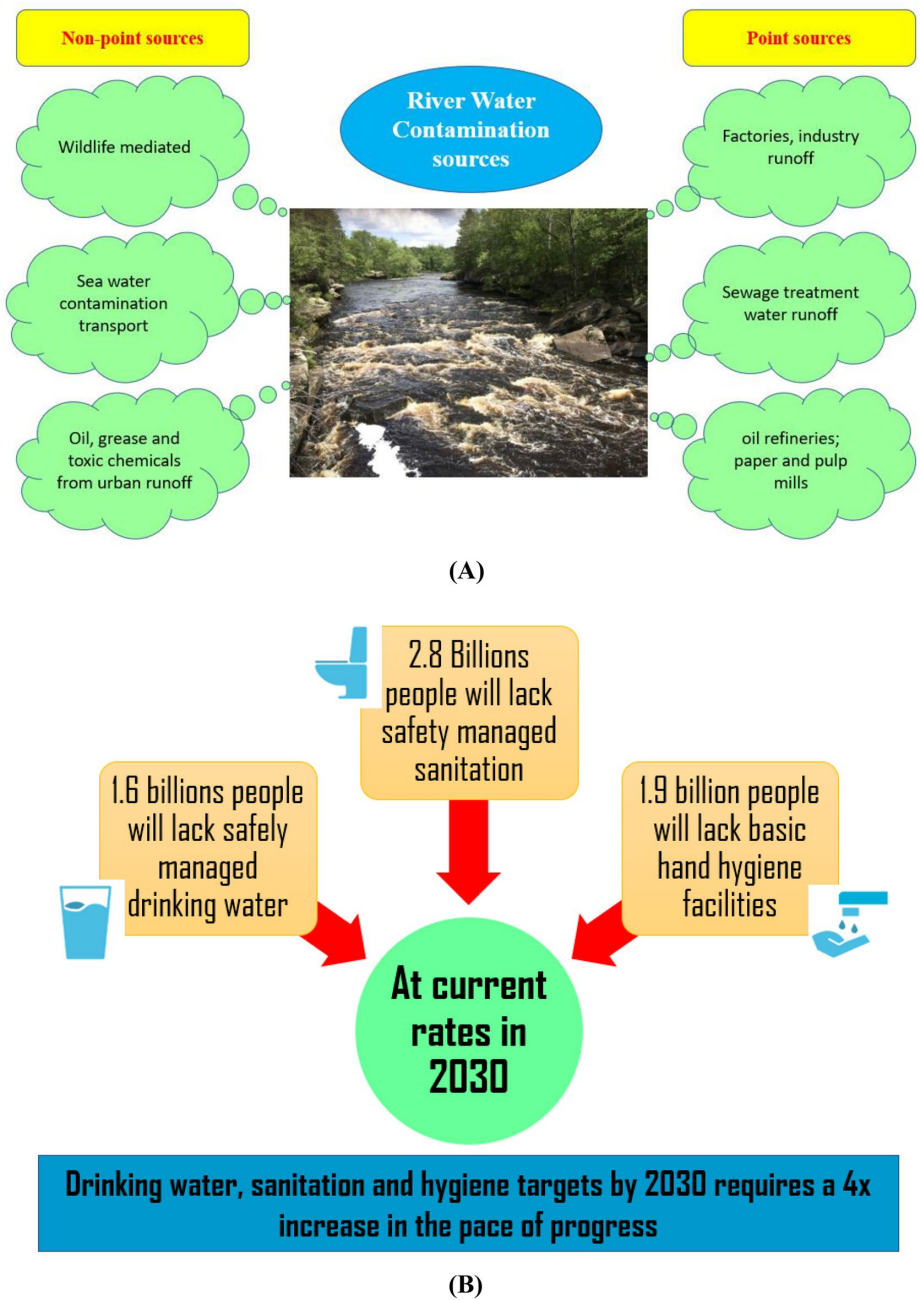
(**A**) Point and nonpoint sources of river water contamination. (**B**) Expectations of sanitation, hygiene, and clean water in 2030.

Rivers, as a notable source of freshwater, represent a valuable natural resource. However, the water quality and quantity have deteriorated owing to the dynamic nature of river bodies and human activities^1,2^. The electrical conductivity (EC), which is a measure of water’s capacity to conduct electrical current, is a key indicator for assessing and identifying compositional changes^3^. A high concentration of dissolved solids typically translates to high EC values^4^. Hence, the EC can be used to identify high salinity levels in irrigation and drinking water when classifying the surface water quality (WQ). The WQ is typically classified based on the sodium content and EC^5^. For seawater and freshwater, the EC is up to 50,000 μS/cm and 0–1500 μS/cm, respectively. The Wilcox EC-based categorization for irrigation water classifies water with EC levels of 0–750 μS/cm as fine, 750–2000 μS/cm as allowable, and > 2000 μS/cm as unacceptable. EC levels higher than 10,000 S/cm are not acceptable for either human consumption or agricultural usage. According to the World Health Organization (WHO, 1993), the recommended maximum EC for drinking water is 1400 $\upmu$S/cm. The WQ classification scheme presented by^6^ is frequently used. In general, the WQ parameters are highly nonlinear, complex, and nonstationary owing to various interconnections with point and nonpoint contamination sources^7,8^ (Fig. 1A). Therefore, the WQ must be accurately predicted, detected, and quantified to ensure the sustainable use and effective management of water resources^9^. In fact, ensuring access to safe water, sanitation, and hygiene (Fig. 1B,^10^ is one of the 17 sustainable development goals for 2030^11^. Given the significance of this goal, environmental engineers must formulate scientific and practical strategies to accomplish the relevant tasks.

Although several approaches have been proposed to quantify WQ over the past three decades^12,13^, it remains challenging to develop a reliable expert prediction system and quantify the WQ using mathematical models^14,15^. The conventional methods for WQ modeling, such as multilinear regression, are linear models that only partially capture nonstationarities and nonlinearities in the environmental dataset because they presume the stationarity of data^9,16^. Furthermore, the classical machine learning (ML) models typically require considerable data, frequent parameter adjustments, and significant reaction time^17,18^. Therefore, such methods are not suitable for WQ prediction or quantification. To address these problems, several ML models for modeling surface WQ parameters have been developed, which do not necessitate complicated algorithms and theory^19–21^, such as kernel models, fuzzy set logic, neural network models, ensemble models, hybrid ML models, decision tree models, and integrative ML data pre-processing models.

ML model development involves several steps, such as data pre-processing, internal parameter tuning, and input feature optimization, and several advancements have been made in the relevant domains^9,22–24^. Notably, the focus of this study is deep learning (DL) models, as a recently developed subset of ML models^25,26^, and their integration with feature input optimization algorithms^27^ to establish a hybrid ML model for river EC prediction. In general, hybrid models can be implemented at either the optimization or prediction stages, depending on their intended use. Hybrid models integrate different optimization strategies and methodologies, thereby resulting in superior modeling accuracies than those achieved using single models. Researchers have highlighted that the key challenge in WQ prediction is the dominant linear-correlation-pattern-based feature extraction. Nonlinear input features affect the prediction capability for nonlinear and nonstationary problems. Considering these aspects, the primary objective of this study is to evaluate the possibility of Boruta feature selection using the XGB technique for identifying the most sensitive related attributes of the target (i.e., EC) variables. A valuable tool for assessing variations in time series, feature selection frameworks can be integrated as a preliminary step in prediction based on ML models to clarify the key features for learning the prediction matrix and provide useful information regarding the physical form of the predictand–predictor relationship.

Previous water quality modeling efforts using conventional statistical and machine learning methods have shown limitations in fully capturing parameters' nonlinear and nonstationary behavior over time. These approaches also require large datasets and frequent tuning, making them unsuitable for real-time predictive needs. There is a need to develop data-driven techniques that can reliably forecast water quality variables several days into the future to support informed decision-making across various sectors reliant on river resources. Accurate multi-step predictions are crucial for optimal water resource planning and management. This study aims to address existing gaps by developing a hybrid machine learning framework integrating Boruta-XGBoost feature selection with a Convolutional Neural Network-Long Short-Term Memory (CNN-LSTM) model. This ensemble methodology leverages the strengths of optimization algorithms and deep sequence learning architectures for water quality modeling.

The objectives of this study can be summarized as follows: (1) Forecast the EC using four ML approaches: convolutional neural network combined with long short-term memory model CNN-LSTM, multi-layer perceptron neural network (MLP), K-nearest neighbor (KNN), and extreme gradient boosting (XGBoost). (2) Optimize the input data using a novel feature selection technique (Boruta combined with XGBoost algorithm). (3) Compare the performances of different models using various statistical metrics and graphical approaches such as scatter plots and Taylor diagrams. (4) Forecast multi-step ahead EC using the model with the best statistical metrics.

## Material and methods

### Study area

#### Albert River

The Albert River is a perennial river located in Queensland in southeast Australia. With a catchment area of 782 square kilometers, this river is located within the Gold Coast and Scenic Rim Region local government regions. The EC data of Albert River were collected from Bromfleet station (145102B), located at − 27.91 °S and 153.11 °E (http://www.bom.gov.au/waterdata/).

#### Barratta Creek

Barratta Creek is located in North Queensland, Australia. The source is located beneath Bunkers Hill in the Leichhardt Range of the Great Dividing Range, and the creek runs north-eastward. The stream continues through largely uninhabited land beyond Woodhouse Mountain, flows virtually parallel to the Haughton River, crosses the Bruce Highway, enters Bowling Green Bay Conservation Park, and empties into Bowling Green Bay at Jerona before joining the Coral Sea. Along its 109-km length, the river drops 224 m. The EC data of Barratta Creek were collected from Barratta Creek at Northcote (119101A) located at − 19.69 °S and 147.17 °E (http://www.bom.gov.au/waterdata/).

**Figure 2:**
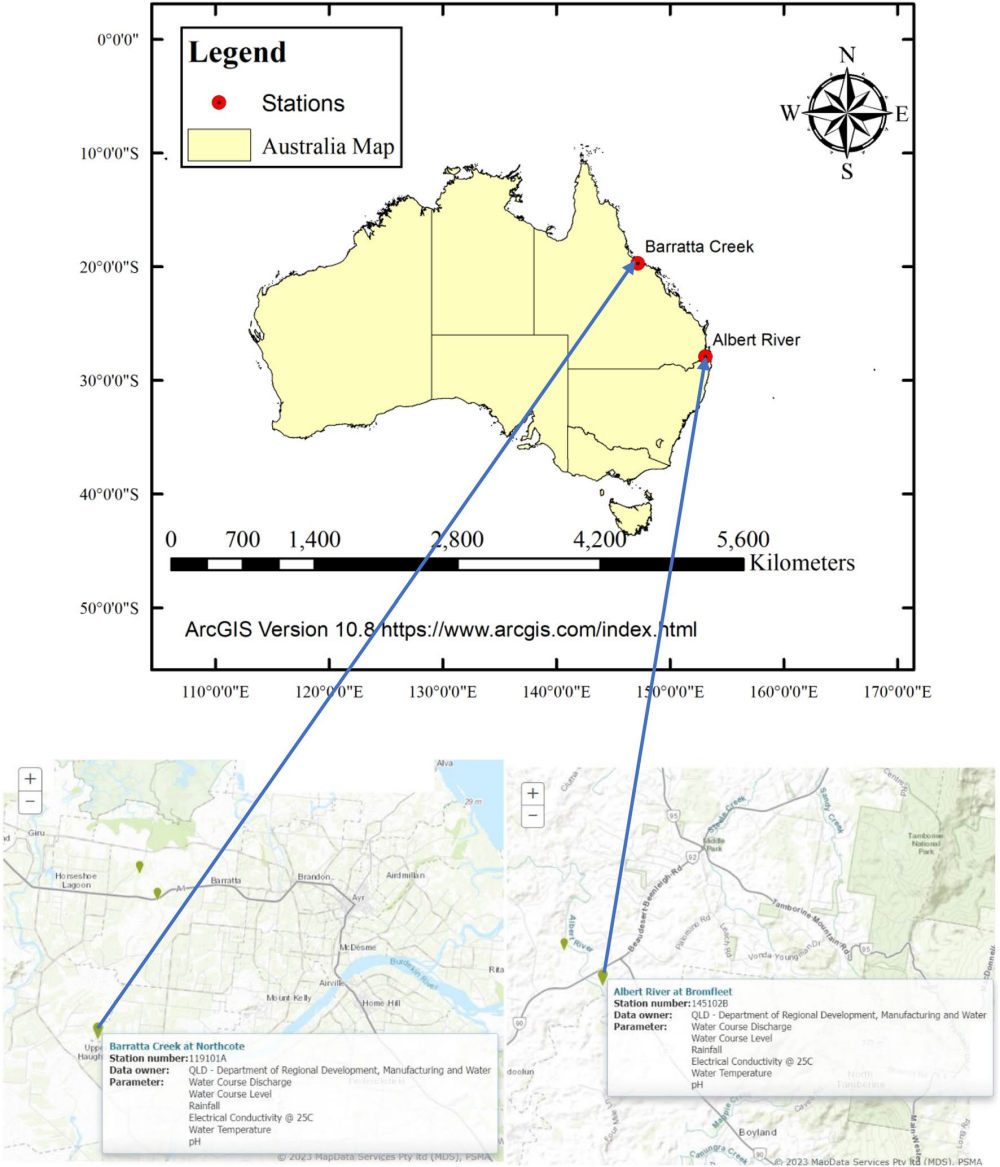
Locations of stations at which the EC was measured (The map was generated using ArcGIS software, version 10.8: https://support.esri.com/zh-cn/products/desktop/arcgis-desktop/arcmap/10-8-1), Australia shape file is from (https://www.abs.gov.au) and maps of River stations are from (http://www.bom.gov.au/waterdata/).

Figure 2 shows the locations of stations at which the EC data were collected.

**Figure 3:**
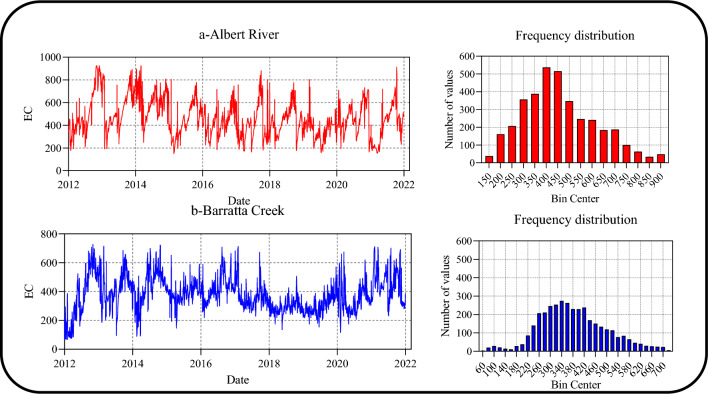
Time series and frequency distributions of EC data for (**a**) Albert River and (**b**) Barratta Creek.

Table 1 summarizes the descriptive statistics of the EC data for both stations. The average observed EC values were 459 and 380 for Albert River and Barratta Creek, respectively. According to the coefficient of variation (C.V) values, the variation in the EC in Albert River (C.V = 34.8%) was larger than that in Barratta Creek (C.V = 31.1%). Figure 3 shows the time series and frequency distribution of the EC values in both stations.

**Table 1:** Descriptive statistics of EC values in the stations of interest (2012–2021).

Metric	Albert River	Barratta Creek
Number of datapoints	3653	3653
Minimum	151	69
Maximum	924	726
Mean	459	380
Median	439	367
Standard deviation	160	118
Coefficient of variation (%)	34.8	31.1
Q1	345	299
Q2	439	367
Q3	563	453
Skewness	0.527	0.314
Kurtosis	− 0.141	0.087

### Boruta-XGBoost feature selection

The Boruta technique is a random forest algorithm wrapper named after the forest god from Slavic mythology^28^ that computes the Z-scores of each predictor's input for the shadow attribute. The major predictor variables are established by the distribution of Z-score metrics^29^. In this study, instead of a random forest, the XGBoost ensemble algorithm was used to calculate the Z-score^30^. The process flow of the Boruta algorithm can be summarized as follows:

1. Random shadow characteristics are created. All data characteristics are shuffled at random, and their numerical order is altered.
2. The XGBoost technique calculates the relevance, expressed by the Z-score, of both the shadow characteristics and original features.
3. The essential characteristics are selected. An original feature with a Z-score greater than the largest Z-score in the set of shadow features is designated as “important”. An original feature with a Z-score considerably lower than that of the shadow features is tagged as “not important” and deleted permanently from the feature set.
4. Steps 1–3 are continued until the significance of all qualities has been marked or the set number of iterations is reached.
5. Details can be found in the work of^31^.

### MLP

MLP, as an architecture of artificial neural networks (ANNs) has been widely employed in various disciplines^32–35^. Similar to other ANN architectures, the MLP receives input signals and processes them before they are transmitted to the other neurons in the hidden layer(s). At least one hidden layer exists in the MLP structure. During the training phase, the neurons in each layer are linked to the neurons in the adjacent layer through a weight. Sigmoid and linear activation functions are typically used in the hidden and output layers to examine the input data characteristics^36^. The MLP can be expressed mathematically as follows:

1 $$y_{MLP} \left( {k + 1} \right) = \mathop \sum \limits_{q} W_{r}^{O} f\left[ {S_{q} \left( k \right)} \right] = \mathop \sum \limits_{q} W_{q}^{O} f\left[ {\mathop \sum \limits_{p} W_{pq}^{I} \left( k \right)x_{p} \left( k \right)} \right]$$

where $q$ is the number of hidden neurons, $x_{p} \left( k \right)$ is the input signal, $S_{q} \left( k \right)$ is the output of the $q$th hidden neuron, and $f$ is the tangent hyperbolic function. The activation function of the output neuron is linear (purelin). Two sets of weights must be updated: those of the input to hidden layer(s), denoted by vector $W^{I} \left( k \right)$), and those of the hidden to output layers, denoted by vector $W^{O} \left( k \right)$. This study adopts MLP networks using a backpropagation algorithm, which can be considered the most prevalent and popular networks. Backpropagation is a supervised learning method that has been used in several prediction tasks^37,38^. In this study, the Levenberg–Marquardt technique, as a backpropagation algorithm, was used to train the MLP network.

### XGBoost

**Figure 4:**
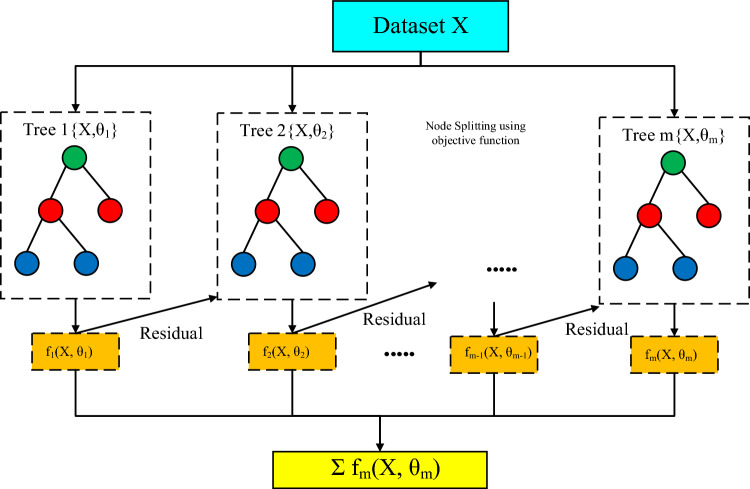
Structure of the XGBoost model.

XGBoost is an improved variant of the gradient boosting tree^39^. Based on the classification and regression tree theory, XGBoost is a successful solution for regression and classification tasks^40–43^. The XGBoost method approximates an objective function (showing the goodness-of-fit) using the quadratic Taylor expansion, enabling more rapid calculations^44^. The core of the algorithm is to optimize the value of the objective function, which typically has two components (training loss and regularization):

2 $${\text{Obj}}\left( {\Theta } \right) = L\left( {\Theta } \right) + {\Omega }\left( {\Theta } \right)$$

where *L* is the loss function of training, and ${\Omega }$ is the regularization term. The training loss is used to evaluate the performance of the model on training data. The regularization term seeks to limit the model complexity, such as overfitting^45^. The complexity can be defined in several ways, with the following expression commonly used for each tree:

3 $${\Omega }\left( f \right) = \gamma T + \frac{1}{2}\lambda \mathop \sum \limits_{j = 1}^{T} \omega_{j}^{2}$$

where *T* is the number of leaves, and $\omega$ represents the vector of leaf scores. The structural score is the following objective function:

4 $${\text{Obj}} = \mathop \sum \limits_{j = 1}^{T} \left[ {G_{j} \omega_{j} + \frac{1}{2}\left( {H_{j} + \lambda } \right)\omega_{j}^{2} } \right] + \gamma T$$

where $\omega_{j}$ are distinct values. The quadratic form $G_{j} \omega_{j} + \frac{1}{2}\left( {H_{j} + \lambda } \right)\omega_{j}^{2}$ is the optimal $\omega_{j}$ for a given structure $q\left( x \right)$. Figure 4 illustrates the structure of the XGBoost model.

### KNN

KNN, developed by^46^, is a well-known ML method for addressing regression and classification problems. The technique includes a variable parameter, k, which represents the number of nearest neighbors. The KNN algorithm operates by locating the data point(s) or neighbors from a training dataset that are the closest to a query point. After selecting the k closest data points, a majority voting rule is applied to determine which class is the most prevalent. The most frequent category is determined to be the final classification for the query. The KNN for regression involves four steps:

1. Determine the distance between the query sample and labeled samples.
  5 $$d\left( {x_{tr} ,x_{t} } \right) = \sqrt {\mathop \sum \limits_{n = 1}^{N} w_{n} \left( {x_{tr,n} - x_{t,n} } \right)^{2} }$$

where $N$ is the number of input features; $x_{tr,n}$ and $x_{t,n}$ are the nth feature values of the training ($x_{tr}$) and testing ($x_{t}$) points, respectively; and $w_{n}$ is the weight of the $n$th feature that ranges between 0 and 1.

2. Arrange the labeled instances in ascending order of the distance.
3. Define the ideal number of neighbors based on the root mean squared error (RMSE), e.g., through cross-validation.
4. Calculate the average distance inversely using the k-nearest neighbors.

### CNN-LSTM

In this study, modern DL techniques are used to develop a prediction model for forecasting the EC in rivers. The CNN-LSTM framework contains two key components: (1) convolutional and pooling layers that perform complicated mathematical operations to produce input data features, (2) LSTM and dense layers that process the obtained features^47^.

#### CNN layer

The one-dimensional CNN (1D-CNN) is a deep feedforward neural network with local connections and weight sharing properties^48^. CNNs can automatically extract high-level dependence characteristics from input data. The learning performance and training duration of the model are determined by its structure, particularly the number of layers. A shallow structure may have inadequate performance, whereas an excessively deep CNN may deteriorate the temporal sequential element of the data or be vulnerable to overfitting^49^.

**Figure 5:**
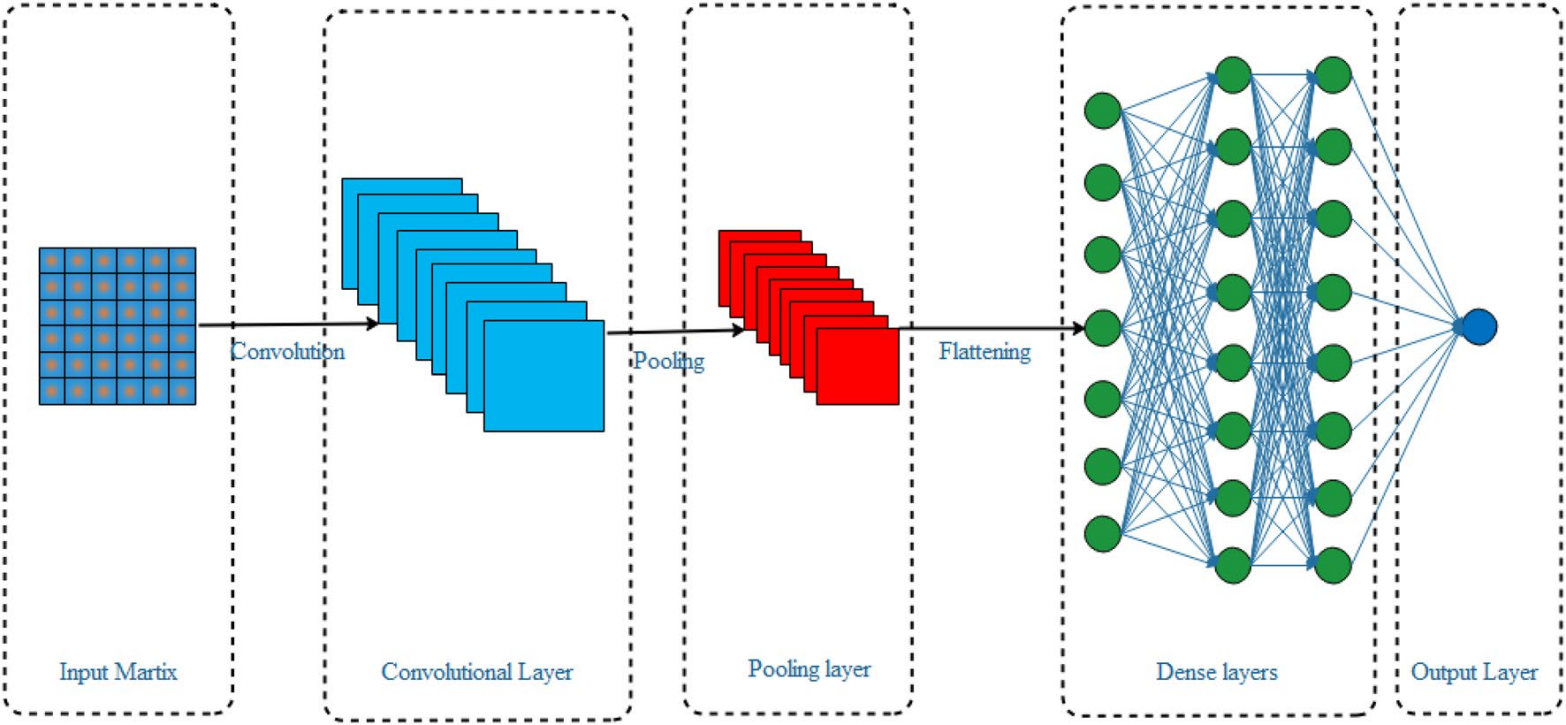
Structure of the CNN deep learning approach.

Typically, the CNN network architecture has convolutional and max-pooling layers^50^. The CNN filter slides along the time axis, and its input is a three-dimensional tensor. The number of CNN convolution kernels is typically determined by the complexity of the objective. A batch normalization layer is added after the convolution layer to enhance the model performance^51^. Overall, CNNs consist of several layers such as the input layer, convolutional layers, nonlinear activation layer, pooling layers, dropout layer, batch normalization layer, one or more completely connected layers, and loss activation layer. Figure 5 shows the structure of the CNN model.

#### LSTM layer

The LSTM is a version of the recurrent neural network: memory blocks composed of memory cells connected by layers, unlike the neurons in ANNs. The approach was proposed by^52^ and improved by^53^ to address the gradient disappearance problem. Each LSTM unit consists of a memory cell and three primary gates: input, output, and forget gates^54^. By determining the information to be forgotten and remembered, the LSTM generates a regulated information flow and learns long-term dependencies. Specifically, the input gate $$i_{t}$$ and a second gate $$c_{t}^{*}$$ control the new information stored in the memory state $$c_{t}$$ at time $$t.$$ The forget gate $$f_{t}$$ regulates the previous information that must be erased or retained on the memory cell at time $$t$$$$-1$$, whereas the output gate $${o}_{t}$$ determines which information may be used to generate the output of the memory cell. Equations (6–10) represent the processes performed by an LSTM unit^55^:

6 $$i_{t} = \sigma \left( {U_{i} x_{t} + W_{i} h_{t - 1} + b_{i} } \right)$$

7 $$f_{t} = \sigma \left( {U_{g} x_{t} + W_{g} h_{t - 1} + b_{g} } \right)$$

8 $$c_{t}^{*} = {\text{tanh}}\left( {U_{c} x_{t} + W_{c} h_{t - 1} + b_{c} } \right)$$

9 $$c_{t} = g_{t} \odot c_{t - 1} + i_{t} \odot c_{t}^{*}$$

10 $$o_{t} = \sigma \left( {U_{o} x_{t} + W_{o} h_{t - 1} + b_{o} } \right)$$

$x_{t }$ represents the input, $W_{*}$ and $U_{{* }}$ are weight matrices, $b_{*}$ represent the bias term vectors, $\sigma$ is the sigmoid function, and $\odot$ represents component-wise multiplication. The output of the memory cell, which is the hidden state h_t_, is computed as

11 $$h_{t} = o_{t} \odot {\text{tanh}}\left( {c_{t} } \right)$$

**Figure 6:**
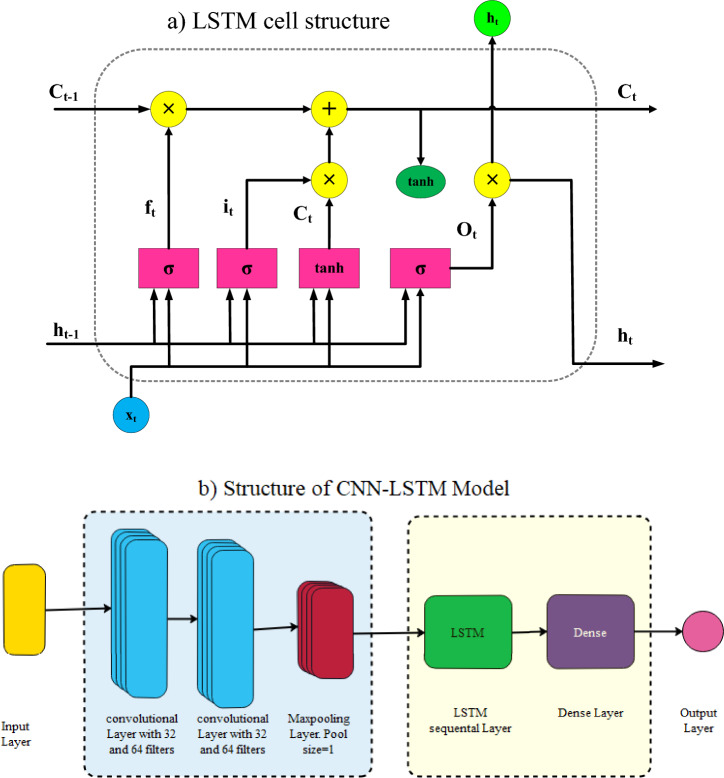
Structures of the (**a**) LSTM cell and (**b**) CNN-LSTM model.

Figure 6 shows the structure of the LSTM cell and CNN-LSTM model that is used to forecast the EC values in rivers.

### Model development

A novel hybrid expert system composed of Boruta-XGBoost as the feature extractor and the CNN-LSTM model was developed to forecast the EC in rivers. Boruta-XGBoost, which is a tree-based feature selection method was used because classical statistical methods such as cross-correlation may introduce lagged time input components with errors owing to the assumption of linearity. Moreover, three other ML models: MLP, XGBoost, and KNN were coupled with the Boruta-XGBoost to validate the main hybrid framework for forecasting the daily EC values in 1-, 3-, 5-, 7-, and 10-month-ahead scenarios for the Barratta Creek and Albert River over the period of 2012 to 2021.

**Figure 7:**
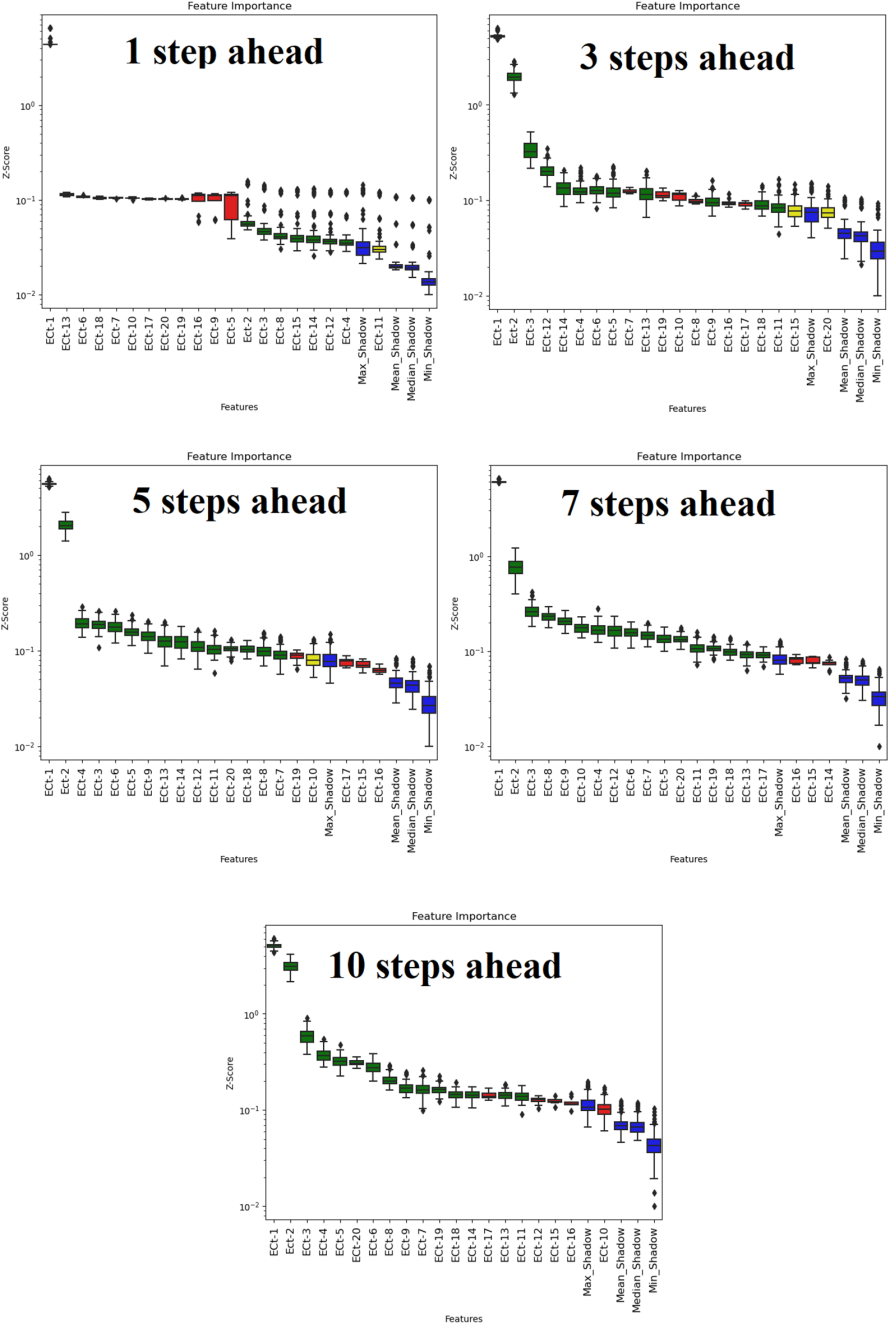
Boruta-XGBoost feature selection results for Albert River EC forecasting.

**Figure 8:**
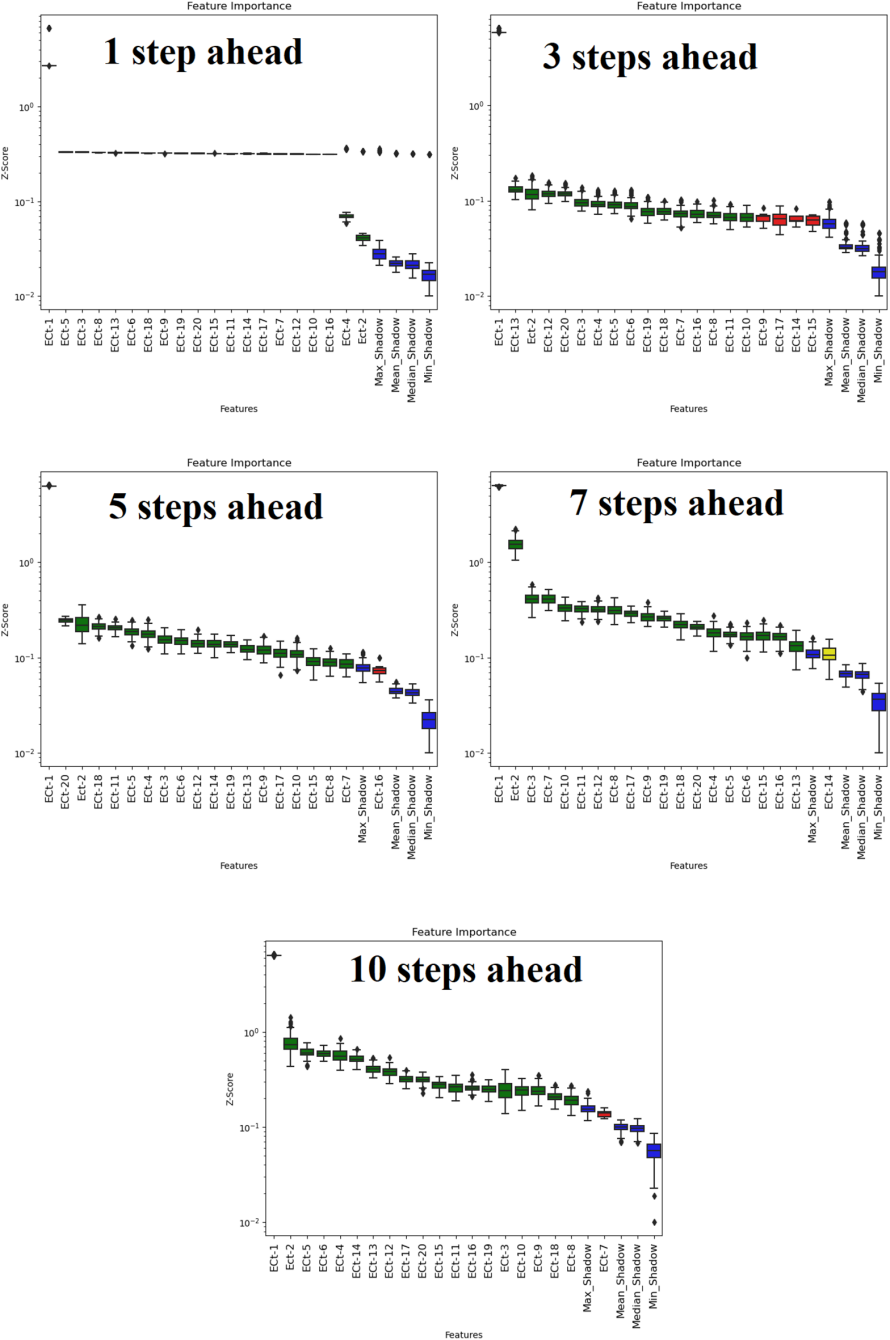
Boruta-XGBoost feature selection results for Barratta Creek EC forecasting.

**Figure 9:**
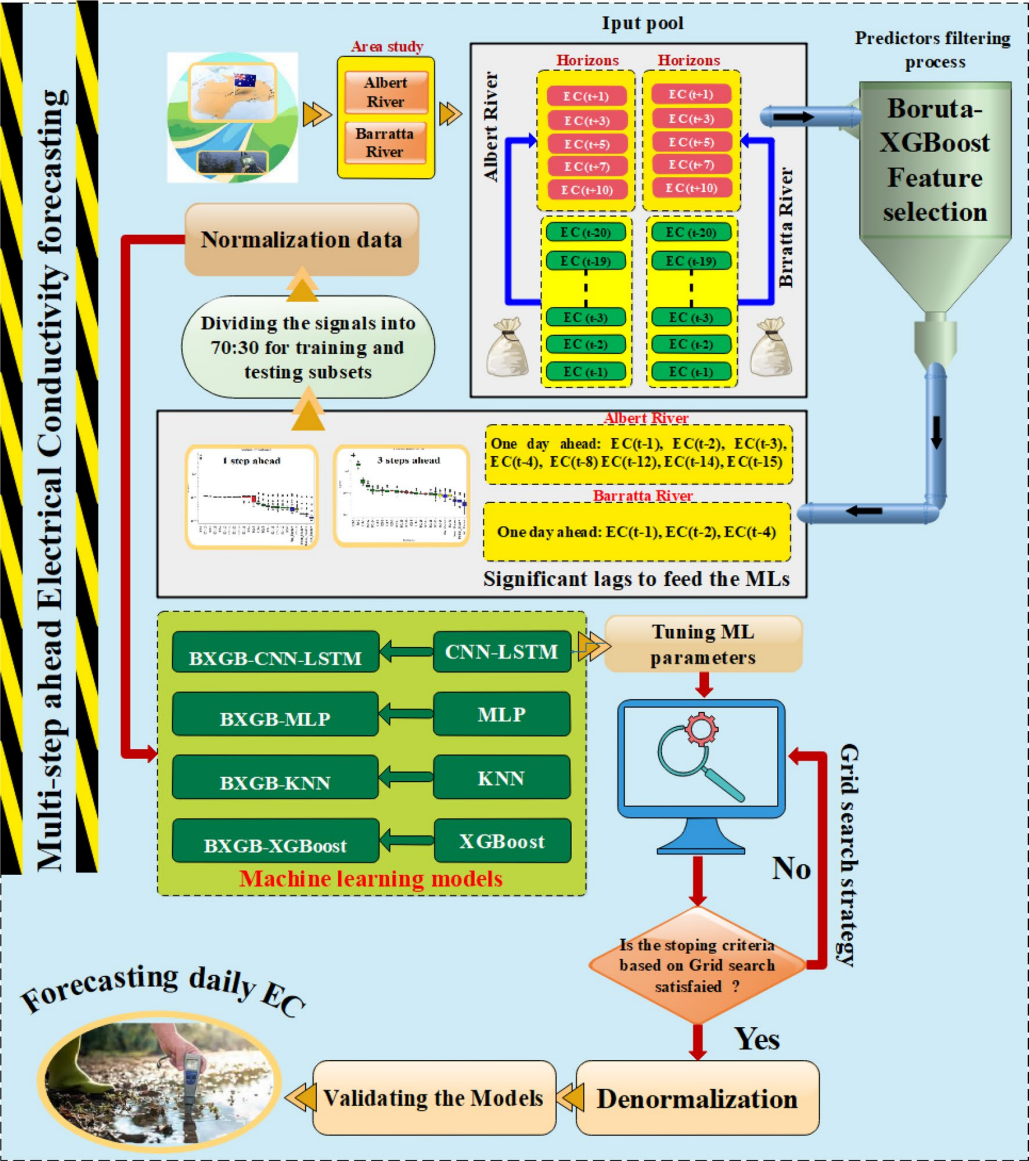
Modeling flowchart of the adopted research.

All the schemes were implemented in Python 3.60, based on the Keras, Scikit-learn, XGBoost, and Boruta-SHAP libraries. Figure 9 shows the process flow of the multi-step forecasting of the EC parameters. As discussed, the Boruta-XGBoost feature selection technique specifies an importance factor for each predictor, i.e., the Z-score^56^. If the Z-score is greater than the max-shadow (a benchmark criterion), the considered predictor is input to the ML models, and the predictors with Z-scores lower than the criterion are ignored^57^. Input pools including 20 lags of EC signals associated with both study areas in four horizons (i.e., 1-day, 3-day, 5-day, 7-day, and 10-day ahead) were assessed using the Boruta-XGBoost approach. Figures 7 and 8 show the results of the Boruta-XGBoost feature selection for the Albert River and Barratta Creek River, respectively. The green predictors are the significant components that pass the max-shadow condition, the red predictors are the rejected entities, and the yellow predictors are tentative entities. Table 2 lists the optimal lagged-time components to be fed to the ML models in the four horizons for each river.

**Table 2:** Significant lags for different time steps.

River	Forecasting (Steps Ahead)	Significant lags
Albert River	1	EC_t-1_, EC_t-2_, EC_t-3_, EC_t-4_, EC_t-8_ EC_t-12_, EC_t-14_, EC_t-15_
	3	EC_t-1_, EC_t-2_, EC_t-3_, EC_t-4_, EC_t-5_, EC_t-6_, EC_t-9_, EC_t-11_, EC_t-12_, EC_t-13_, EC_t-14_, EC_t-18_
	5	EC_t-1_, EC_t-2_, EC_t-3_, EC_t-4_, EC_t-5_, EC_t-6_, EC_t-7_, EC_t-8_, EC_t-9_, EC_t-11_, EC_t-12_, EC_t-13_, EC_t-14_, EC_t-18_, EC_t-20_
	7	EC_t-1_, EC_t-2_, EC_t-3_, EC_t-4_, EC_t-5_, EC_t-6_, EC_t-7_, EC_t-8_, EC_t-9_, EC_t-10_, EC_t-11_, EC_t-12_, EC_t-13_, EC_t-14_, EC_t-17_, EC_t-18_, EC_t-19_, EC_t-20_
	10	EC_t-1_, EC_t-2_, EC_t-3_, EC_t-4_, EC_t-5_, EC_t-6_, EC_t-7_, EC_t-8_, EC_t-9_, EC_t-10_, EC_t-11_, EC_t-13_, EC_t-14_, EC_t-17_, EC_t-18_, EC_t-19_, EC_t-20_
Barratta River	1	EC_t-1_, EC_t-2_, EC_t-4_
	3	EC_t-1_, EC_t-2_, EC_t-3_, EC_t-4_, EC_t-5_, EC_t-6_, EC_t-7_, EC_t-8_ EC_t-10_, EC_t-11_, EC_t-12_, EC_t-13_, EC_t-16_, EC_t-18_, EC_t-19_, EC_t-20_
	5	EC_t-1_, EC_t-2_, EC_t-3_, EC_t-4_, EC_t-5_, EC_t-6_, EC_t-7_, EC_t-8_, EC_t-9_, EC_t-10_, EC_t-11_, EC_t-12_, EC_t-13_, EC_t-14_, EC_t-15_, EC_t-17_, EC_t-18_, EC_t-19_, EC_t-20_
	7	EC_t-1_, EC_t-2_, EC_t-3_, EC_t-4_, EC_t-5_, EC_t-6_, EC_t-7_, EC_t-8_, EC_t-9_, EC_t-10_, EC_t-11_, EC_t-12_, EC_t-13_, EC_t-15_, EC_t-16_, EC_t-17_, EC_t-18_, EC_t-19_, EC_t-20_
	10	EC_t-1_, EC_t-2_, EC_t-3_, EC_t-4_, EC_t-5_, EC_t-6_, EC_t-8_, EC_t-9_, EC_t-10_, EC_t-11_, EC_t-12_, EC_t-13_, EC_t-14_, EC_t-15_, EC_t-16_, EC_t-17_, EC_t-18_, EC_t-19_, EC_t-20_ EC_t-19_, EC_t-20_

It is necessary to use an appropriate strategy for splitting the time-series dataset for forecasting. Generally, approximately 60–80% of the dataset is used for training the models, and the rest is used for validation. To this end, cross-validation strategies such as k-fold cross-validation^58^, holdout, and walking-forward^59^ approaches are promising to avoid overfitting. In this study, the holdout strategy was used, with 70% and 30% of the dataset used for training and testing, respectively.

Four powerful ML models were used to forecast the daily EC: Boruta-XGB-MLP, Boruta-XGB-XGBoost, Boruta-XGB-KNN, and Boruta-XGB-CNN-LSTM (proposed). Notably, the hyperparameters in hybrid models must be appropriately tuned to avoid overfitting while obtaining the optimal modeling results. To this end, various free-source strategies such as grid search, random search, and Bayesian optimization can be applied and implemented in various programming languages such as MATLAB and Python^60,61^. In this research, the ML model is optimized using the grid search technique. Table 3 summarizes the optimal settings, network architecture, and hyperparameters associated with the four ML models. The key hyperparameters of the Boruta-XGB-CNN-LSTM approach, as the model of interest, were the number of LSTM layers number, number of CNN layers, number of neurons, training algorithm, and learning rate^62^.

**Table 3:** Model adjustment for EC forecasting.

Study site	Models	Parameters
Albert	Boruta-XGB-MLP	Layers: 1, Number of Neurons: 7, Training Algorithm: Levenberg–Marquardt
	Boruta-XGB-XGBoost	N_Estimators: 80, Max-Depth: 4, Learning Rate: 0.1
	Boruta-XGB-KNN	N_Neighbors: 4, Weights: ('uniform')
	Boruta-XGB-CNN-LSTM	CNN Layers: 2, Filters: 32,32, Kernel Size: 3,3, Pool_Size:1, LSTM Layer: 1; Number of Neurons: 90, Dense Layer: 1, Number of Neurons: 70, Learning Rate: 0.0017, Training Algorithm: Adam, Batch Size: 64, Epochs: 34
Barratta	Boruta-XGB-MLP	Layers: 1, Number of Neurons: 5, Training Algorithm: Levenberg–Marquardt
	Boruta-XGB-XGBoost	N_Estimators: 100, Max-Depth: 6, Learning rate: 0.15
	Boruta-XGB-KNN	N_Neighbors: 5, Weights = ('uniform')
	Boruta-XGB-CNN-LSTM	CNN Layers: 2, Filters: 64,64, kernel size: 2,2, Pool_Size:1, LSTM Layer: 1, Number of Neurons: 60, Dense Layer: 1, Number of Neurons: 100, Learning Rate: 0.0005, Training Algorithm: Adam, Batch Size: 64, Epochs: 45

		Multi-step Ahead
Albert	3	CNN Layers: 2, Filters: 32,32, Kernel Size: 3,3, Pool_Size: 1, LSTM Layer: 1, Number of Neurons: 90, Dense Layer: 1, Number of Neurons: 70, Learning Rate: 0.001, Training Algorithm: Adam, Batch Size: 64, Epochs: 29
	5	CNN Layers: 2, Filters: 32,32, Kernel Size: 3,3, Pool_Size: 1, LSTM Layer: 1, Number of Neurons: 80, Dense Layer: 1, Number of Neurons: 80, Learning Rate: 0.0015, Training Algorithm: Adam, Batch Size: 32, Epochs: 30
	7	CNN Layers: 2, Filters: 32,32, Kernel Size: 3,3, Pool_Size:1, LSTM Layer: 1, Number of Neurons: 90, Dense Layer: 1, Number of Neurons: 12, Learning Rate: 0.0012, Training Algorithm: Adam, Batch Size: 64, Epochs: 47
	10	CNN Layers: 2, Filters: 32,32, Kernel Size: 3,3, Pool_Size: 1, LSTM Layer: 1, Number of Neurons: 100, Dense Layer: 1, Number of Neurons: 100, Learning Rate: 0.00125, Training Algorithm: Adam, Batch Size: 64, Epochs: 33
Barratta	3	CNN Layers: 2, Filters: 32,32, Kernel Size: 3,3, Pool_Size: 2, LSTM Layer: 1 Number of Neurons: 100, Dense Layer:1, Number of Neurons: 100, Learning Rate: 0.025, Training Algorithm: Adam, Batch Size: 64, Epochs: 37
	5	CNN Layers: 2, Filters: 32,32, Kernel Size: 3,3, Pool_Size: 2, LSTM Layer: 1, Number of Neurons: 100, Dense Layer:1, Number of Neurons: 100, Learning Rate: 0.01, Training Algorithm: Adam, Batch Size: 64, Epochs: 50
	7	CNN Layers: 2, Filters: 32,32, Kernel Size: 3,3, Pool_Size: 2, LSTM Layer: 1, Number of Neurons: 100, Dense Layer: 1, Number of Neurons: 100, Learning Rate: 0.006, Training Algorithm: Adam, Batch Size: 64, Epochs: 40
	10	CNN Layers: 2, Filters: 32,32, Kernel Size: 3,3, Pool_Size: 1, LSTM Layer: 1, Number of Neurons: 100, Dense Layer: 1, Number of Neurons: 100, Learning Rate: 0.004, Training Algorithm: Adam, Batch Size: 64, Epochs: 29

A pre-processing step, classical normalization, was applied to mitigate the negative effects of the data scale: All the inputs and targets were limited between zero and one. This operation is typically applied to increase the rate of convergence and modeling accuracy^63^.

### Statistical metrics

Six statistical indices were used evaluate the robustness of the ML models: RMSE, correlation coefficient (R), uncertainty with a confidence level of 95% ($U_{95\% }$), mean absolute percentage error (MAPE), T-statistic test ($T_{stat}$), and Nash–Sutcliffe model efficiency coefficient (NSE)^60,61^, expressed as follows:

12 $$R = \frac{{\mathop \sum \nolimits_{{{\text{i}} = 1}}^{{\text{N}}} \left( {EC_{o,i } - \overline{{EC_{o } }} } \right){ }\left( {EC_{p,i} { } - { }\overline{{EC_{p} }} } \right){ }}}{{\sqrt {\mathop \sum \nolimits_{{{\text{i}} = 1}}^{{\text{N}}} (EC_{o,i } - \overline{{EC_{o } }} )^{2} { }\mathop \sum \nolimits_{{{\text{i}} = 1}}^{{\text{N}}} (EC_{p,i} { } - { }\overline{{EC_{p} }} )^{2} { }} { }}}$$

13 $$RMSE = \sqrt {\frac{1}{N} \mathop \sum \limits_{i = 1}^{N} \left( {EC_{o,i } - EC_{p,i} } \right)^{2} }$$

14 $$MAPE = \frac{1}{N}\mathop \sum \limits_{i = 1}^{N} \left| {\frac{{EC_{o,i } - EC_{p,i} }}{{EC_{o,i } }}} \right| \times 100$$

15 $$E = 1 - \frac{{\mathop \sum \nolimits_{i = 1}^{N} \left( {EC_{o,i} - EC_{p,i} } \right)^{2} }}{{\mathop \sum \nolimits_{i = 1}^{N} \left( {EC_{o,i} - \overline{EC}_{o} } \right)^{{2^{2} }} }}$$

16 $$T_{stat} = \sqrt {\frac{{\left( {N - 1} \right)MBE^{2} }}{{RMSE^{2} - MBE^{2} }}}$$

17 $$U_{95\% } = 1.96\sqrt {SD_{e}^{2} + RMSE^{2} }$$

where $EC_{o,i }$ and $EC_{p,i}$ are the measured and forecasted values of EC, respectively; $\overline{{EC_{o } }}$ and $\overline{{EC_{p} }}$ are the mean measured and forecasted values of EC, respectively; MBE is the mean bias error; and N is the length of the time series. The best and worst fitting between the measured and forecasted values of the EC occurs correspond to the following values: (R = 1, $E$ = 1, MAPE = 0, RMSE = 0, and $U_{95\% }$ = 0) and (R = 1, $E$ = $- \infty$, MAPE = $\infty$, RMSE = $\infty$, and $U_{95\% }$ = $\infty$), respectively^64,65^.

### Ethical approval

The manuscript is conducted in the ethical manner advised by the water resource management journal.

### Consent to publish

The research is scientifically consented to be published.

## Results and discussion

The forecasting ability of the Boruta-XGB-CNN-LSTM, Boruta-XGB-MLP, Boruta-XGB-KNN, and Boruta-XGB-XGBoost models for multi-step ahead EC for the two Australian rivers was evaluated using the evaluation metrics (R, RMSE, MAPE, E, Tstat, and U_95%_) for the training and testing stages and diagnostic plots.

Table 4 presents the one-step ahead forecasting results of the four models for the Albert River at Bromfleet. The Boruta-XGB-CNN-LSTM model outperformed the other models in the training (R = 0.9515, RMSE = 51.2558, MAPE = 5.9893, E = 0.9032, Tstat = 7.5962, U_95%_ = 141.2955) and testing (R = 0.9429, RMSE = 45.6896, MAPE = 5.9749, E = 0.8878, Tstat = 3.3426, U_95%_ = 126.3533) periods in the one-step-ahead EC forecasting for the Albert River. Boruta-XGB-XGBoost exhibited the second-best performance, followed by Boruta-XGB-MLP and Boruta-XGB-KNN based on the goodness-of-fit metrics.

**Table 4:** Results of one-step ahead EC forecasting for the Albert River.

Model	Data	R	RMSE	MAPE	E	Tstat	U_95%_
Boruta-XGB-CNN-LSTM	Train	0.9515	51.2558	5.9893	0.9032	7.5962	141.2955
	Test	0.9429	45.6896	5.9749	0.8878	3.3426	126.3533
Boruta-XGB-MLP	Train	0.9508	51.1386	6.1955	0.9037	1.0253	141.7482
	Test	0.9261	52.7775	9.2756	0.8503	0.4877	146.3171
Boruta-XGB-KNN	Train	0.9631	44.5649	5.7544	0.9268	4.8466	123.2562
	Test	0.8302	82.4990	19.1113	0.6342	0.3056	228.7228
Boruta-XGB-XGBoost	Train	0.9635	44.1885	5.5588	0.9281	0.1263	122.4963
	Test	0.9323	52.4448	9.5378	0.8522	1.8652	145.2876

**Figure 10:**
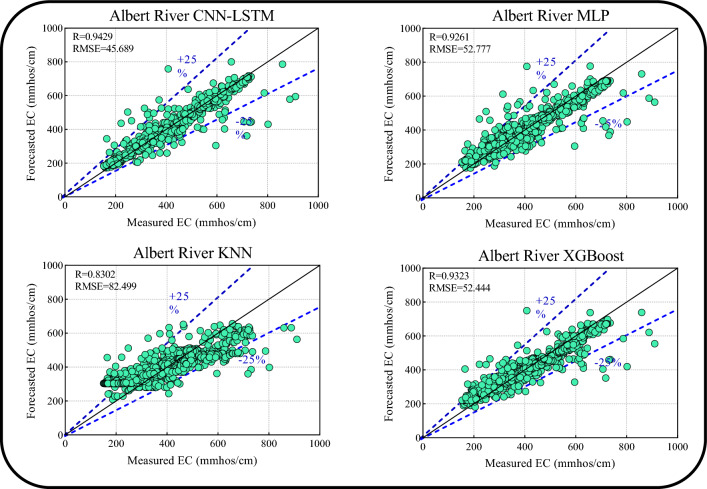
Scatter plots of forecasted versus measured EC for the Albert River.

Figure 10 shows the scatter plots for the Boruta-XGB-CNN-LSTM and comparative models, incorporating the upper and lower bounds, in terms of the R and RMSE metrics between the measured and forecasted one-step-ahead EC (Albert River) in the testing period. The Boruta-XGB-CNN-LSTM model exhibited the highest accuracy with R = 0.9429 and RMSE = 45.68, followed by XGBoost (R = 0.9323 and RMSE = 52.444), MLP (R = 0.9261 and RMSE = 52.777), and Boruta-XGB-KNN (R = 0.8302 and RMSE = 82.499). Furthermore, the forecast generated by the Boruta-XGB-CNN-LSTM model lay within the 25% upper and lower bound thresholds, indicating a strong relationship between the forecasted and measured EC.

**Figure 11:**
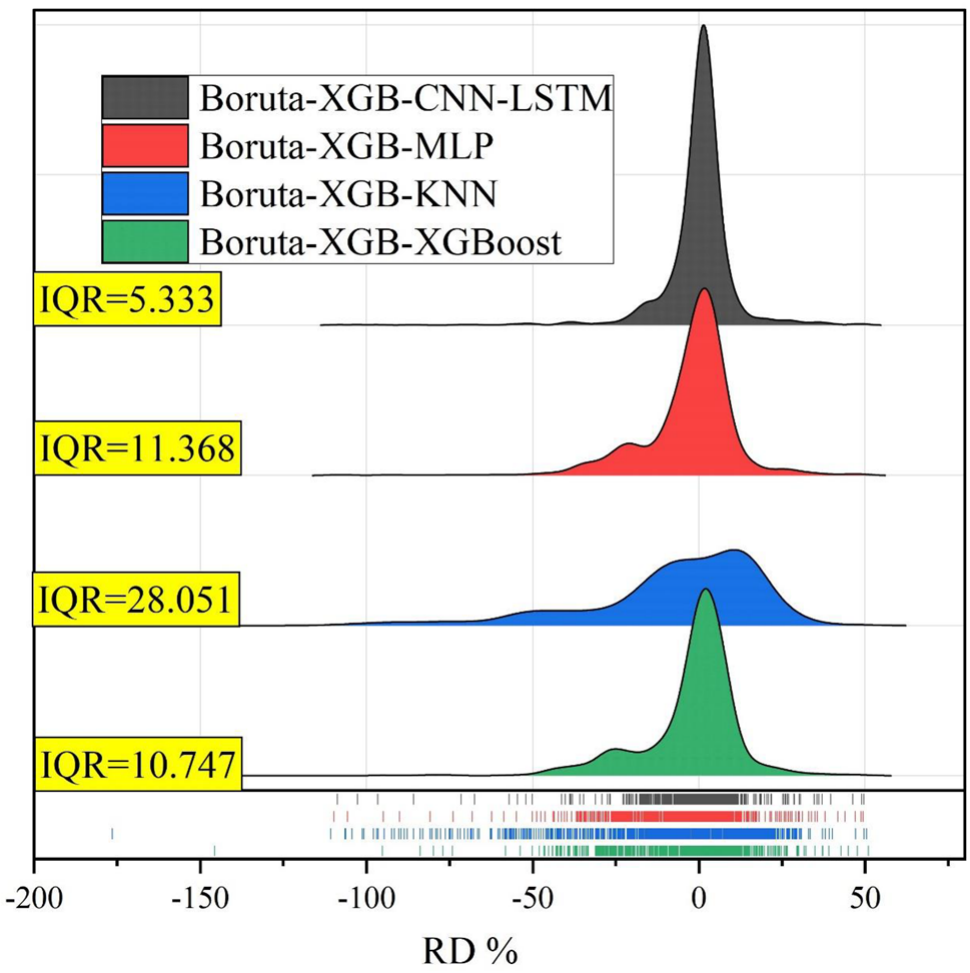
Ridge plots of relative deviation percent (RD %) for the Albert River EC forecasted by different models.

Figure 11 shows the ridge plots, which indicate the relative deviation percent (RD, %) to assess the one-step-ahead EC forecasts for the Albert River obtained by the Boruta-XGB-CNN-LSTM and comparative models. In addition, the interquartile range (IQR) values are presented. The Boruta-XGB-CNN-LSTM model produced the most accurate RD distribution with the lowest IQR = 5.333. The benchmark Boruta-XGB-XGBoost model was superior to the Boruta-XGB-MLP and Boruta-XGB-KNN model.

Table 5 presents the one-step ahead forecasting results of the four models for Barratta Creek. The proposed Boruta-XGB-CNN-LSTM model was slightly more accurate than the comparative models in the training period (R = 0.9316, RMSE = 43.2172, MAPE = 7.6428, E = 0.8673, Tstat = 2.7861, U_95%_ = 119.7122) and testing period (R = 0.9215, RMSE = 43.8315, MAPE = 7.6029, E = 0.8488, Tstat = 1.1701, U_95%_ = 121.4845). Although the performance of the comparative models was satisfactory, it was lower than that of the proposed approach in forecasting the one-step ahead EC for Barratta Creek.

**Table 5:** Results of one-step ahead EC forecasting for Barratta Creek.

Model	Data	R	RMSE	MAPE	E	Tstat	U_95%_
Boruta-XGB-CNN-LSTM	Train	0.9316	43.2172	7.6428	0.8673	2.7861	119.7122
	Test	0.9215	43.8315	7.6029	0.8488	1.1701	121.4845
Boruta-XGB-MLP	Train	0.9288	44.1936	7.9495	0.8616	3.8830	122.3293
	Test	0.9184	44.7175	7.7053	0.8426	2.5231	123.7993
Boruta-XGB-KNN	Train	0.9443	39.0323	7.4576	0.8918	0.1975	108.2023
	Test	0.9042	48.3154	8.7854	0.8162	1.4046	133.8936
Boruta-XGB-XGBoost	Train	0.9546	35.4029	6.8040	0.9110	0.0785	98.1415
	Test	0.9128	46.0644	8.5283	0.8330	0.9702	127.6856

**Figure 12:**
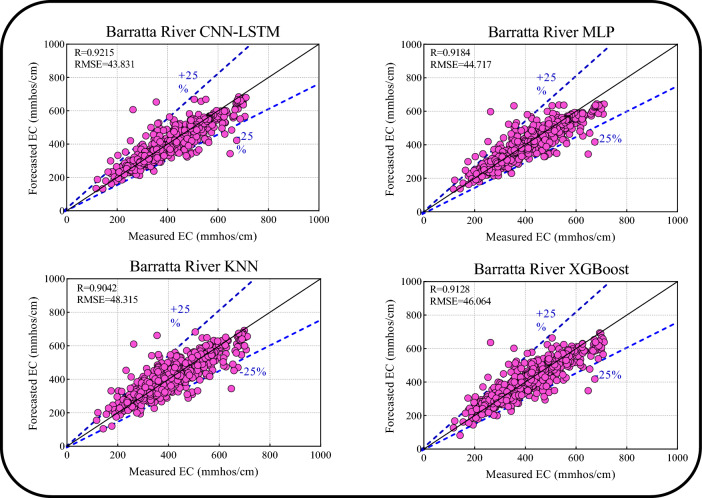
Scatter plots of forecasted versus measured EC values for Barratta Creek.

Figure 12 shows the scatter plots for the Boruta-XGB-CNN-LSTM and comparative models, incorporating the upper and lower bounds, in terms of the R and RMSE metrics between the measured and forecasted one-step-ahead EC (Barratta Creek). The Boruta-XGB-CNN-LSTM model achieved the highest accuracy (R = 0.9215 and RMSE = 43.831), and the forecast lay within the 25% range between the upper and lower bound thresholds. The models ranking second, third, and fourth in terms of the accuracy were Boruta-XGB-MLP (R = 0.9184 and RMSE = 44.717), Boruta-XGB-XGBoost (R = 0.9128 and RMSE = 46.064), and Boruta-XGB-KNN (R = 0.9042 and RMSE = 48.315), respectively. Although the 25% upper and lower bounds were reasonable for the comparative models, the Boruta-XGB-CNN-LSTM was the best model in this forecasting task.

**Figure 13:**
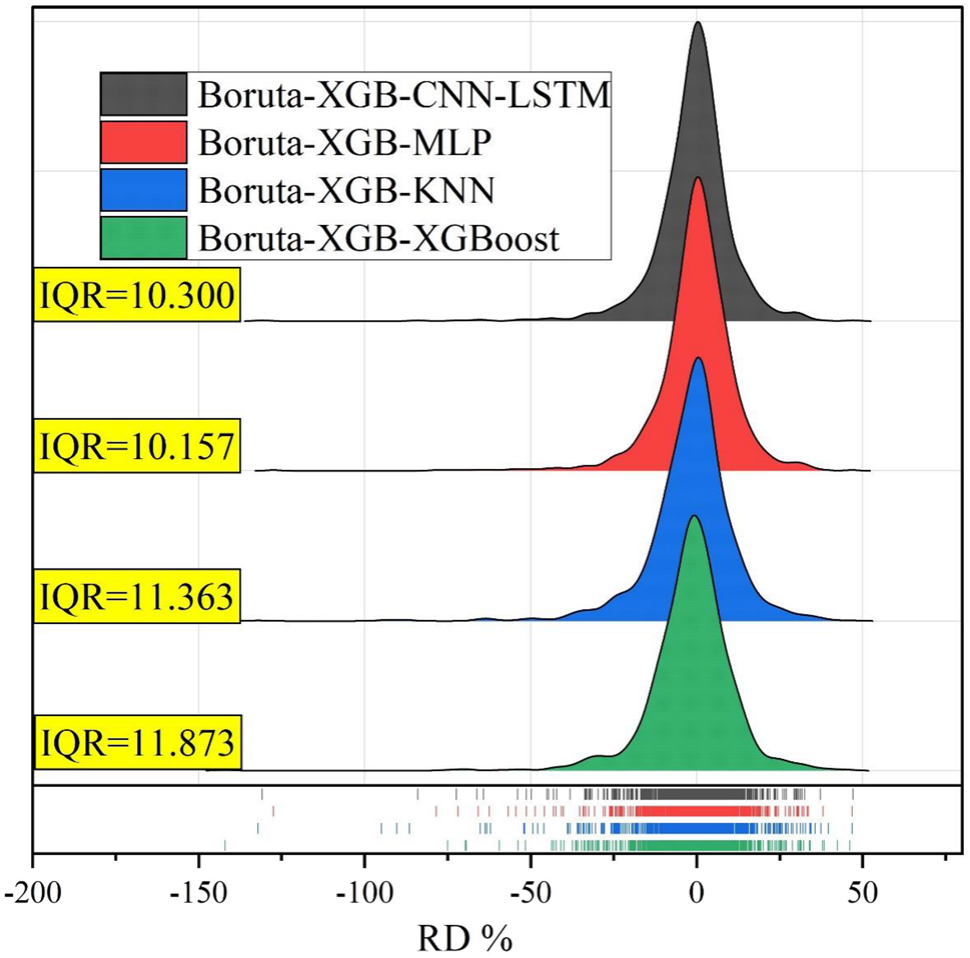
Ridge plots of RD (%) for the Barratta Creek EC forecasted by different models.

Figure 13 shows the ridge plots for Barratta Creek to indicate the RD (%) errors and IQR values. Although all models exhibit reasonable RD (%) errors, the forecasts based on the Boruta-XGB-CNN-LSTM model are slightly more accurate with IQR = 10.30, followed by Boruta-XGB-MLP (IQR = 10.157), Boruta-XGB-KNN (IQR = 11.363), and Boruta-XGB-XGBoost (IQR = 11.873). Therefore, the proposed model yields the most accurate one-step-ahead EC forecasts for Barratta Creek.

**Figure 14:**
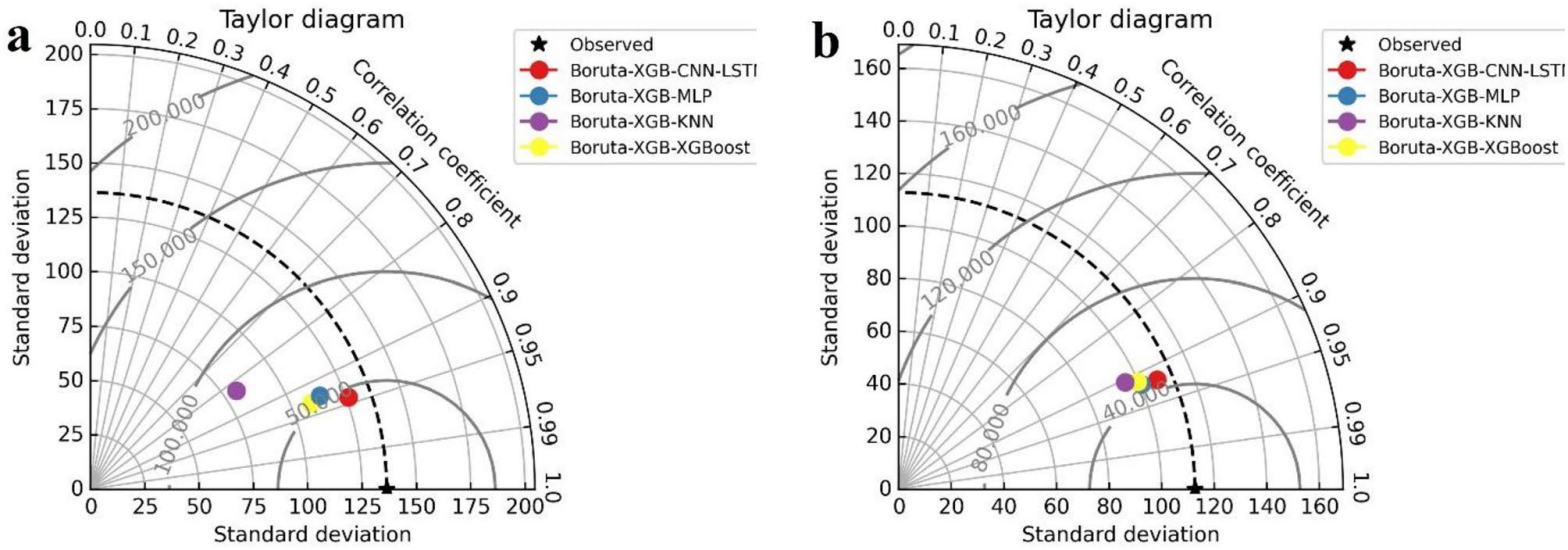
Taylor diagram for one-step-ahead EC forecasting for (**a**) Albert River and (**b**) Barratta River.

Figure 14 shows the Taylor diagram of the one-step-ahead EC forecasted by the Boruta-XGB-CNN-LSTM, MLP, KNN, and XGBoost models for (A) Albert River and (B) Barratta Creek. The Taylor diagram is a valuable tool for comprehensively assessing the model's comparability against the observed EC using the standard deviation and correlation coefficient. For Albert River, the Boruta-XGB-CNN-LSTM (blue solid circle) forecast was close to the measured EC, with a correlation coefficient of more than 0.95 and standard deviation ranging between 125 and 150. The Boruta-XGB-MLP, Boruta-XGB-KNN, and Boruta-XGB-XGBoost predictions were slightly far from the measured EC with a correlation coefficient lower than 0.95 and standard deviation ranging between 100 and 150. For Barratta Creek, the Boruta-XGB-CNN-LSTM (red solid circle) model exhibited the highest precision with a correlation coefficient of 0.90–0.95, followed by the Boruta-XGB-MLP, Boruta-XGB-XGBoost, and Boruta-XGB-KNN models. In other words, the Boruta-XGB-CNN-LSTM model was superior in forecasting the one-step ahead EC for both Albert River and Barratta Creek.

### Multi-step ahead forecasting

Table 6 presents the metrics for the Boruta-XGB-CNN-LSTM multi-step ahead forecasts (i.e., 3-, 5-, 7-, and 10-day-ahead) EC for Albert River. The forecasting accuracy in the 3-day-ahead scenario was higher than that for the 7- and 10-day-ahead cases in both the training and testing periods, as indicated by the superior goodness-of-fit metrics for the 3-day-ahead forecasts: (R = 0.8947, RMSE = 73.6800, MAPE = 10.4113, E = 0.7998, Tstat = 2.3851, U_95%_ = 204.1362) for the training period and (R = 0.8764, RMSE = 66.3651, MAPE = 12.0275, E = 0.7633, Tstat = 4.7504, U_95%_ = 183.0642) for the testing period. Similarly, the 5-day-ahead was superior to that of the 7- and 10-day-ahead forecasts but inferior to that of the 3-day-ahead horizon. In other words, the proposed model attained a higher precision in short-term forecasting (i.e., 1-, 3-, and 5-day) compared with that for long-term forecasting (i.e., 7- and 10-day) of the EC for Albert River.

**Table 6:** Results of multi-step ahead EC forecasting for the Albert River.

Horizon	Data	R	RMSE	MAPE	E	Tstat	U_95%_
3-steps-ahead	Train	0.8947	73.6800	10.4113	0.7998	2.3851	204.1362
	Test	0.8764	66.3651	12.0275	0.7633	4.7504	183.0642
5-steps-ahead	Train	0.8674	81.9761	12.8438	0.7521	1.8250	227.1739
	Test	0.8326	75.6831	14.0573	0.6922	1.4061	209.7361
7-steps-ahead	Train	0.8404	90.1850	15.0560	0.6994	6.1947	249.0694
	Test	0.7934	83.1531	16.8054	0.6284	0.1597	230.5399
10-steps-ahead	Train	0.8004	98.5680	17.4178	0.6402	0.2013	273.2424
	Test	0.7367	92.7799	20.2669	0.5374	3.5644	256.4918

**Figure 15:**
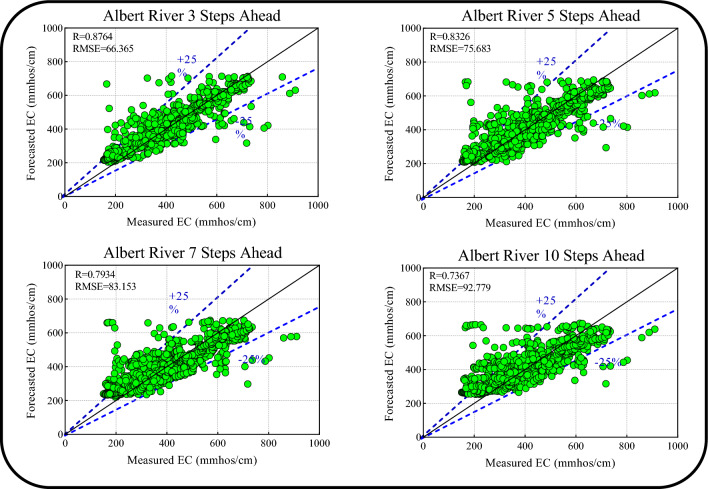
Scatter plots of multi-step ahead forecasted EC versus measured EC for the Albert River.

Figure 15 shows the scatterplots along with the R and RMSE metrics of the Boruta-XGB-CNN-LSTM model for multi-step ahead (i.e., 3-, 5-, 7-, and 10-day) EC forecasts for the Albert River. In addition, the 25% upper and lower bound confidence intervals are presented. The strongest correlation is observed for the 3-day-ahead EC forecasts, given the highest R (0.8764) and lowest RMSE (66.365), although the forecasts for the 5-, 7-, and 10-day-ahead EC forecasts are also satisfactory. Overall, the proposed model is better at short-term EC forecasting (1-, 3-, and 5-day), and the performance decreases over long-term forecast horizons (i.e., 7- and 10-day) for the Albert River.

**Figure 16:**
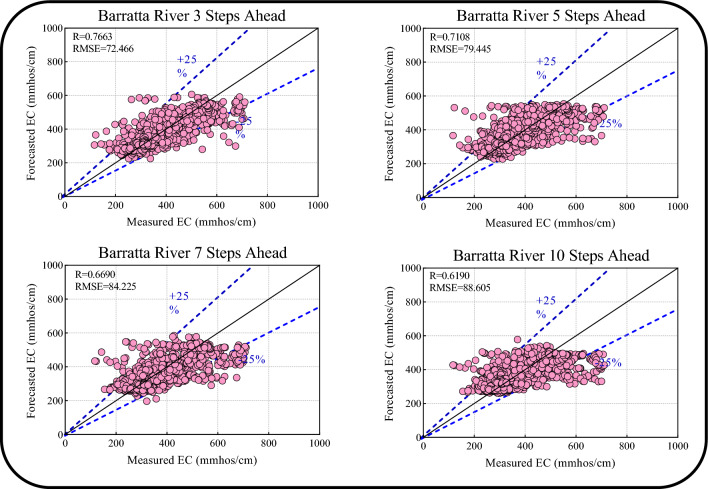
Scatter plots of multi-step ahead forecasted EC versus measured EC for the Barratta Creek.

Table 7 and Fig. 16 present the multi-step ahead (i.e., 3-, 5-, 7-, and 10-day) EC forecasts for Barratta Creek obtained using the proposed Boruta-XGB-CNN-LSTM model. Table 7 shows that the model yields more accurate forecasts in the 3- and 5-day-ahead horizon compared with the 7- and 10-day-ahead horizons in the training and testing periods. This finding is supported by the scatter plots in Fig. 16. The short-term forecasts (3- and 5-day-ahead) are more accurate (R of 0.7677 and 0.7108, respectively) with lower RMSEs (72.466 and 79.445, respectively) compared with those of the 7- and 10-day-ahead horizons. Therefore, the Boruta-XGB-CNN-LSTM model is more effective for short-term EC forecasting in Barratta Creek station.

**Table 7:** Results of multi-step ahead EC forecasting for Barratta Creek.

Horizon	Data	R	RMSE	MAPE	E	Tstat	U95
3-steps-ahead	Train	0.8339	65.6887	14.9481	0.6929	2.2169	182.0097
	Test	0.7663	72.4667	14.7316	0.5866	0.8251	200.8821
5-steps-ahead	Train	0.7878	72.9874	16.5071	0.6195	2.7618	202.1789
	Test	0.7108	79.4455	16.5628	0.5032	1.7305	220.1116
7-steps-ahead	Train	0.7651	76.1118	16.7717	0.5852	1.0503	210.9690
	Test	0.6690	84.2257	16.7168	0.4416	2.6831	233.1329
10-steps-ahead	Train	0.7314	81.0510	17.3607	0.5284	5.6692	223.9778
	Test	0.6190	88.6057	18.0405	0.3820	1.4281	245.5441

## Discussion

The results demonstrate the effectiveness of the proposed Boruta-XGB-CNN-LSTM model in accurately forecasting EC for the Albert River and Barratta Creek across different time horizons. For predictions from one day ahead, the hybrid model outperformed other ML approaches according to multiple statistical evaluation metrics. This indicates the benefits of optimizing input features and leveraging CNN-LSTM architectures for water quality prediction. Notably, short-term forecasts up to 5 days achieved higher accuracy than longer 7–10-day horizons. This is understandable, given the increasing uncertainty for more distant predictions. However, the model still produced reasonably good accuracy even 10 days ahead, suggesting usefulness for supporting various planning functions. While performance decreased with lead time as expected, the slight deterioration demonstrates the model's ability to learn dependencies beyond immediate observations. This capacity to capture rich temporal patterns should aid in addressing non-stationarities in environmental systems. Comparing performance across stations reveals the approach is transferable despite rivers' differing characteristics. Tests on independent sites within Australia indicate potential for applicability in diverse settings pending location-specific tuning. The study’s findings have several potential applications and implications for improving water resource management and environmental monitoring. The accurate multi-step electrical conductivity forecasts produced by the Boruta-XGB-CNN-LSTM model allow river authorities to optimize water allocation and reservoir operations over different timescales. This helps balance the needs of water users. The model's predictions also help pollution control agencies identify at-risk areas and implement targeted mitigation strategies. Meanwhile, drinking water facilities and industries can better treat incoming supplies if alerted in advance about changing EC levels via the forecasts. Agricultural producers and fish farmers could also utilize the projections to schedule irrigation and select suitable crops/species. Furthermore, the predictions may aid emergency responders during flood and contamination events. Overall, systematically incorporating data-driven insights enables the development of long-term, sustainable river basin management strategies while considering both current and future water quality conditions. The reliable, AI-powered monitoring and forecasting capabilities also support compliance with environmental regulations over time.

## Conclusion

A hybrid CNN-LSTM model was used to forecast multi-step ahead EC for the Albert River and Barratta Creek in Australia. The proposed model was optimized using the Boruta-XGBoost algorithm to rank and select the best input features. Forecasting was performed over the 1-, 3-, 5-, 7-, and 10-day horizons to demonstrate the applicability of the Boruta-XGB-CNN-LSTM model. Moreover, the forecasting performance of the proposed method was compared with those of the state-of-the-art models: Boruta-XGB-MLP, Boruta-XGB-XGBoost, and Boruta-XGB-KNN. The goodness-of-fit metrics demonstrated that the hybrid Boruta-XGB-CNN-LSTM could effectively forecast the multi-step ahead EC for both rivers. In particular, the proposed model attained the highest precision in the testing period for the Albert River (R = 0.9429, RMSE = 45.6896, MAPE = 5.9749, E = 0.8878, Tstat = 3.3426, U_95%_ = 126.3533) and Barratta Creek (R = 0.9215, RMSE = 43.8315, MAPE = 7.6029, E = 0.8488, Tstat = 1.1701, U_95%_ = 121.4845) in forecasting one-step ahead EC. Moreover, the Boruta-XGB-CNN-LSTM was more accurate in short-term (i.e., 1-, 3-, and 5-day) forecasting, and its performance slightly deteriorated in the 7- and 10-day-ahead forecast horizons. The proposed model can be extended to other applications such as agriculture, environmental, and atmospheric modeling.

While the proposed Boruta-XGB-CNN-LSTM model achieved good performance, some limitations still exist. The study utilized daily water quality and meteorological data from only two rivers within Australia, so expanding data collection from more diverse locations globally would help validate the generalizability and robustness of models. Additionally, additional real-time data sources like satellite imagery could help capture spatial influences and improve forecasts. The study focused on predicting a single water quality parameter but developing multi-parameter models that simultaneously forecast other important indices would increase practical relevance. Moreover, while measures were taken to prevent overfitting, more rigorous validation techniques like uncertainty quantification on out-of-sample data could provide a realistic assessment of long-term forecast accuracy. Addressing these limitations through multidisciplinary collaborations in future work would help advance the development of widely applicable AI solutions for integrated water resource and ecosystem management globally.

## Data Availability

Data sets generated during the current study are available from the corresponding author on reasonable request.
