# Publisher Correction: Multi-step ahead forecasting of electrical conductivity in rivers by using a hybrid Convolutional Neural Network-Long Short-Term Memory (CNN-LSTM) model enhanced by Boruta-XGBoost feature selection algorithm

**DOI:** 10.1038/s41598-024-69309-3

**Published:** 2024-08-09

**Authors:** Masoud Karbasi, Mumtaz Ali, Sayed M. Bateni, Changhyun Jun, Mehdi Jamei, Aitazaz Ahsan Farooque, Zaher Mundher Yaseen

**Affiliations:** 1 Canadian Centre for Climate Change and Adaptation, University of Prince Edward Island, St Peters Bay, PE Canada; 2 Water Engineering Department, Faculty of Agriculture, University of Zanjan, Zanjan, Iran; 3 UniSQ College, University of Southern Queensland, Springfield Campus, QLD 4301 Australia; 4 Department of Civil, Environmental and Construction Engineering and Water Resources Research Center, University of Hawaii at Manoa, Honolulu, HI 96822 USA; 5 Department of Civil and Environmental Engineering, College of Engineering, Chung-Ang University, Seoul, Republic of Korea; 6 Faculty of Civil Engineering and Architecture, Shahid Chamran University of Ahvaz, Ahvaz, Iran; 7 Faculty of Sustainable Design Engineering, University of Prince Edward Island, Charlottetown, PE C1A4P3 Canada; 8 Civil and Environmental Engineering Department, King Fahd University of Petroleum & Minerals, 31261 Dhahran, Saudi Arabia; 9 New Era and Development in Civil Engineering Research Group, Scientific Research Center, Al-Ayen University, Thi-Qar, Nasiriyah 64001 Iraq

Correction to: *Scientific Reports* 10.1038/s41598-024-65837-0, published online 01 July 2024

In the original version of this Article, Changhyun Jun and Aitazaz Ahsan Farooque were omitted as corresponding authors. The correct corresponding authors for this Article are Masoud Karbasi, Changhyun Jun and Aitazaz Ahsan Farooque.

The original Article has been corrected.

